# Unenhanced CT as an Alternative to Contrast-Enhanced CT in Evaluating Renal Cryoablation Zones

**DOI:** 10.7759/cureus.71295

**Published:** 2024-10-12

**Authors:** Hiroki Yano, Hiroki Higashihara, Yasushi Kimura, Yukihiro Enchi, Soichi Nakamura, Hiroki Satomura, Yuji Koretsune, Kaishu Tanaka, Yusuke Ono, Noriyuki Tomiyama

**Affiliations:** 1 Department of Diagnostic and Interventional Radiology, Osaka University Graduate School of Medicine, Suita, JPN; 2 Department of High Precision Image-Guided Percutaneous Intervention, Osaka University Graduate School of Medicine, Suita, JPN; 3 Department of Radiology, Osaka University Hospital, Suita, JPN

**Keywords:** contrast enhanced ct, ct guided ethanol ablation, non-contrast-enhanced ct, percutaneous cryoablation, renal cell carcinoma (rcc), unenhanced ct

## Abstract

Background

Advances in imaging technology and the increased use of abdominal imaging have led to a rise in renal cell carcinoma (RCC) detection. While surgery remains the primary treatment for small RCCs, minimally invasive procedures like cryoablation are gaining popularity, particularly for patients with comorbidities or renal dysfunction. CT-guided cryoablation offers advantages, including high spatial resolution and real-time visualization during the procedure. Post-procedure imaging is essential for assessing treatment success, with contrast-enhanced CT (CE-CT) typically considered vital. However, many patients, especially older individuals, have renal dysfunction that limits the use of contrast agents. In such cases, unenhanced CT (UE-CT) presents a viable alternative for post-procedural evaluation. This study explored the effectiveness of UE-CT in assessing cryoablation zones as a substitute for CE-CT.

Materials and Methods

This retrospective study included 54 patients (58 tumors) who underwent cryoablation at a single institution between 2014 and 2024. Only patients with available early follow-up CT (within three days post-cryoablation) and subsequent follow-up were included. Tumors marked with lipiodol prior to cryoablation and cases requiring transcatheter arterial embolization due to extravasation immediately after cryoablation were excluded. Percutaneous renal cryoablation was performed under CT fluoroscopy, and the ablation zone was assessed using a 64-channel multi-slice CT scanner. UE-CT was conducted before the procedure, followed by both UE-CT and CE-CT within three days after cryoablation. CT attenuation values were measured for pre-procedure UE-CT (kidneys and tumor), post-procedure UE-CT (kidneys, cryoablation zone, and tumor), and post-procedure CE-CT (kidneys, cryoablation zone, and tumor). Tumor volumes in the post-procedure regions were evaluated on both UE-CT and CE-CT. Statistical analyses were performed using Wilcoxon’s signed-rank test and Spearman’s rank correlation coefficient, with interobserver agreement determined by the intraclass correlation coefficient.

Results

The median tumor diameter was 1.56 cm (IQR: 1.33-2.00 cm). On UE-CT, the cryoablation zone exhibited high attenuation, while it showed low attenuation on CE-CT. The median attenuation values of the kidneys on UE-CT before and after cryoablation were not significantly different (33.6 Hounsfield unit (HU) vs. 34.3 HU, P = 0.17). However, on CE-CT, the median attenuation values of normal kidneys and the cryoablation zone significantly differed (171.7 HU vs. 55.7 HU, P < 0.0001). Similarly, on UE-CT, there was a significant difference in the median attenuation values between normal kidneys and the cryoablation zone (34.3 HU vs. 47.4 HU, P < 0.0001). The median renal volumes of the unenhanced regions on CE-CT and those with attenuation changes on UE-CT were not significantly different (26.52 cm³ vs. 28.83 cm³, P = 0.86). These values showed a strong correlation (r = 0.95; 95% CI: 0.91-0.97).

Conclusions

This study showed that UE-CT can reliably estimate the ablation zone in RCC patients post-cryoablation. While the contrast between the ablation zone and normal renal parenchyma was lower on UE-CT compared to CE-CT, the ablation zone was still detectable and highly correlated with CE-CT results. Further research with larger sample sizes is needed to validate the clinical utility of UE-CT and assess the reproducibility of these findings.

## Introduction

The detection of renal cell carcinoma (RCC) has increased in recent years due to advancements in imaging technology and the more frequent use of abdominal imaging [1,2]. Surgery remains the primary treatment for small RCC, while minimally invasive procedures have emerged as alternatives for patients with comorbidities or renal dysfunction [3]. Cryoablation has become an increasingly popular treatment option for small RCCs worldwide [4,5]. CT guidance provides several advantages, including superior spatial resolution and the ability to visualize changes during cryoablation [6,7]. Accurate post-procedure imaging is essential for assessing treatment adequacy and guiding further management.

While contrast-enhanced CT (CE-CT) is typically considered crucial for evaluating the ablation zone [8], many patients, particularly older ones, have renal dysfunction that limits the use of contrast agents. In such cases, unenhanced CT (UE-CT) is an ideal alternative for post-procedural evaluation. However, the effectiveness of UE-CT in evaluating the cryoablation zone remains unclear. Therefore, this study investigated the utility of UE-CT for assessing the ablation zone.

## Materials and methods

Patient selection

**Figure 1:**
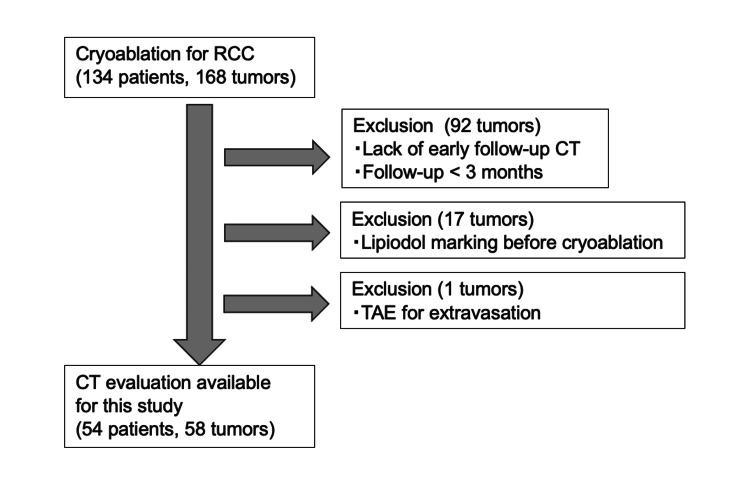
Study flowchart This flowchart illustrates the study’s patient selection process. Patients who lacked early follow-up CT or had follow-up periods of less than three months (92 tumors), those with lipiodol marking prior to cryoablation (17 tumors), and patients who underwent TAE for extravasation (one tumor) were excluded. The final cohort for CT evaluation included 54 patients with 58 tumors. RCC, renal cell carcinoma; TAE, transcatheter arterial embolization

This retrospective study was approved by the Institutional Review Board (Ethical Review Board of Osaka University Hospital), with informed consent waived (IRB no. 18445-2). A total of 128 patients who underwent cryoablation for 148 RCC lesions between 2014 and 2024 were included. In all cases, a renal mass biopsy was performed either before or during treatment. Each case was managed in consultation with both a urologist and an interventional radiologist. Of the lesions, 72 tumors were excluded due to the absence of early follow-up CT imaging (within three days post-cryoablation) or discontinuation of follow-up. Seventeen tumors marked with lipiodol prior to cryoablation were also excluded, as the retained lipiodol significantly impacted CT evaluation. Additionally, one tumor requiring transcatheter arterial embolization (TAE) due to extravasation immediately following cryoablation was excluded. The study flowchart is presented in Figure 1.

Renal cryoablation technique

Renal cryoablation was performed percutaneously using a cryoablation system (Galil Medical, Yokneam, Israel) under CT fluoroscopy guidance (Siemens Healthineers, Erlangen, Germany). The placement of the probes and the puncture angle were determined based on the tumor size using preoperative CT imaging. Organ protection techniques, such as artificial pneumothorax, hydrodissection, and warm saline injection via nephrostomy, were employed as necessary. The cryoablation procedure consisted of two freeze-thaw cycles.

CT evaluation of the ablation zone

A 64-channel multi-slice CT scanner (Discovery CT 750 HD; GE Healthcare, Chicago, IL, USA) was utilized for post-cryoablation imaging. For the enrolled patients, UE-CT was conducted prior to the procedure, followed by both UE-CT and CE-CT within three days after cryoablation (Day 2: n = 50; Day 3: n = 4). Attenuation values were evaluated for pre-procedure UE-CT (kidneys and tumor), post-procedure UE-CT (kidneys, cryoablation zone, and tumor), and post-procedure CE-CT (kidneys, cryoablation zone, and tumor). The tumor volume was assessed after the procedure using both UE-CT (cryoablation zone and tumor) and CE-CT (cryoablation zone and tumor).

All regions were measured in three dimensions using VINCENT software (version 6.7.0007; Fujifilm, Tokyo, Japan) by two certified diagnostic radiologists. Initially, the attenuation values of normal kidneys were analyzed for changes from baseline following cryotherapy. Subsequently, the attenuation values of the normal kidneys and the cryoablation zone, along with their differences, were measured using both UE-CT and CE-CT. Finally, the volume of the cryoablation zone was quantified by both UE-CT and CE-CT, using the median difference in attenuation values as the threshold.

Statistical analysis

Statistical analyses were conducted using EZR (version 4.2.1; Saitama Medical Center, Jichi Medical University, Saitama, Japan) and Excel (Microsoft Corporation, Redmond, WA, USA). Variables between the two groups were compared using Wilcoxon’s signed-rank test and Spearman’s rank correlation coefficient, as appropriate. All parameters were assessed by two diagnostic radiologists, and the average value was utilized. The intraclass correlation coefficient (ICC) was calculated to evaluate interobserver agreement. A p-value of less than 0.05 was considered statistically significant. Correlation strengths were classified as follows: very weak (r < 0.19), weak (r = 0.20-0.39), moderate (r = 0.40-0.59), strong (r = 0.60-0.79), and very strong (r = 0.80-1.00). Data were presented as the median and IQR unless otherwise specified.

## Results

The patient and tumor characteristics are presented in Table 1.

**Table 1:** Patient and tumor characteristics eGFR, estimated glomerular filtration rate

Variables	Patients (n = 54)
Mean age, years	75 (62-81)
Sex	
Male	43
Female	11
Surgical history of the kidney	
Nephrectomy	15
Partial nephrectomy	6
Autologous kidney transplant	1
eGFR, mL/min/1.73 m²	53.5 (44.5-64.9)
Creatinine, mg/dL	1.03 (0.87-1.25)
Primary disease	
Von Hippel-Lindau disease	4
Tumor laterality	
Left	29
Right	29
Tumor diameter, cm	1.56 (1.33-2.00)

**Figure 2:**
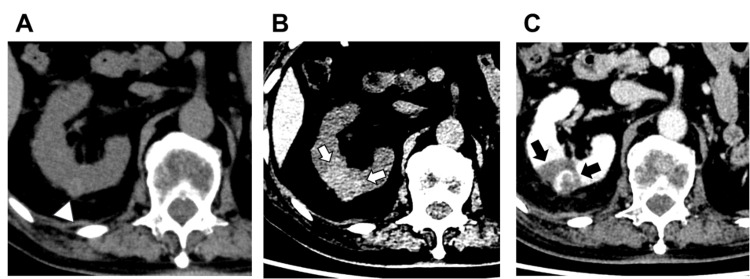
Axial CT images before and after cryoablation for RCC (A) Pre-procedure UE-CT shows the tumor (indicated by the white arrowhead) in the right kidney. (B) Post-cryoablation UE-CT illustrates the ablation zone, marked by the white arrows, which demonstrates higher attenuation. (C) Post-cryoablation CE-CT reveals the boundary (indicated by black arrows) with lower attenuation, further confirming the ablation zone. CE-CT, contrast-enhanced CT; RCC, renal cell carcinoma; UE-CT, unenhanced CT

In total, the study included 58 tumors (median diameter: 1.56 cm (IQR = 1.33-2.00); range: 0.59-3.56 cm) across 54 patients (43 men and 11 women; median age: 75 years (IQR = 62-81); range: 23-91 years). All tumors were classified as cT1a stage RCC. Representative CT images are shown in Figure 2.

The cryoablation zone displayed high attenuation on UE-CT and low attenuation on CE-CT. The ICC for intra-observer agreement is presented in Table 2.

**Table 2:** Intra-observer agreement of CT parameters CE-CT, contrast-enhanced CT; ICC, intraclass correlation coefficient; UE-CT, unenhanced CT

Intra-observer agreement of measurement	ICC (2, 1)
CT values	
Pre-procedure kidney (UE-CT)	0.95
Post-procedure kidney (UE-CT)	0.96
Post-procedure cryoablation zone (UE-CT)	0.95
Post-procedure kidney (CE-CT)	0.73
Post-procedure cryoablation zone (CE-CT)	0.96
Ablation zone volume	
Post-procedure cryoablation zone (UE-CT)	0.65
Post-procedure cryoablation zone (CE-CT)	0.61

**Figure 3:**
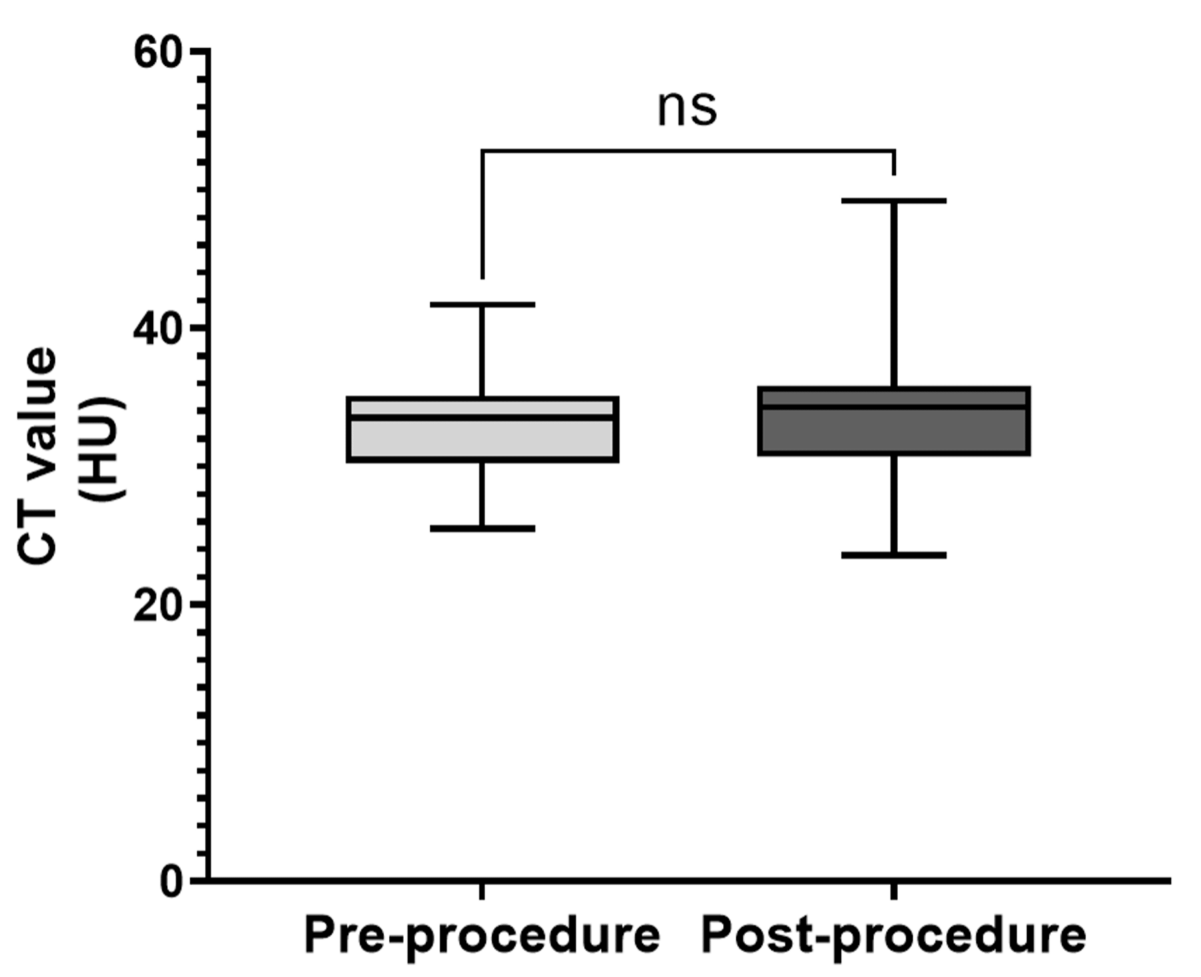
Attenuation values for normal kidneys on UE-CT before and after cryoablation The median attenuation values for the kidneys on UE-CT before and after cryotherapy were 33.5 HU (IQR = 30.3-35.1) and 36.7 HU (IQR = 31.4-44.7), respectively. No significant difference was observed between the two groups (P = 0.17). HU, Hounsfield unit; ns, not significant; UE-CT, unenhanced CT

The median attenuation values for the kidneys on UE-CT before and after cryotherapy were 33.6 Hounsfield unit (HU; IQR = 30.4-35.1) and 34.3 HU (IQR = 30.8-35.8), respectively (P = 0.17; Figure 3).

**Figure 4:**
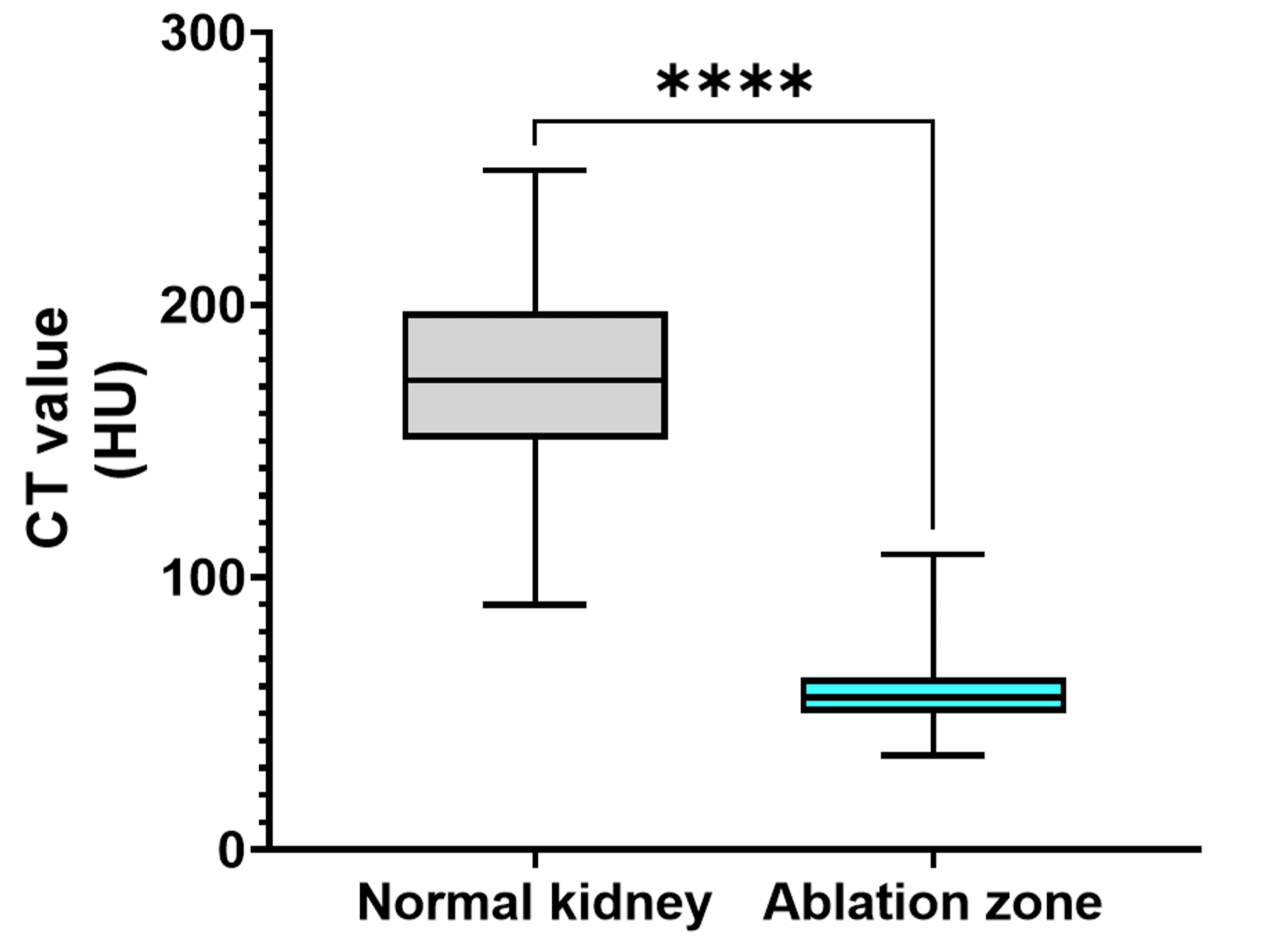
Attenuation values for normal kidneys and the ablation zone following cryoablation on CE-CT The median attenuation values for normal kidneys and the cryoablation zone were 172.5 HU (IQR = 150.9-196.8) and 55.9 HU (IQR = 50.8-62.8), respectively (P < 0.0001). CT, contrast-enhanced CT; HU, Hounsfield unit

On CE-CT, the median attenuation values for normal kidneys and the cryoablation zone were 171.7 HU (IQR = 151.8-196.8) and 55.7 HU (IQR = 50.5-62.8), respectively (P < 0.0001; Figure 4).

**Figure 5:**
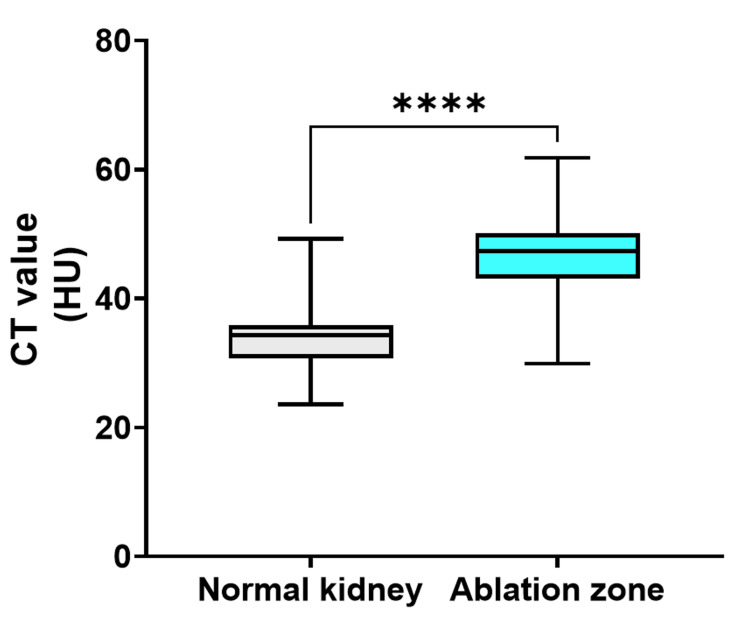
Attenuation values for normal kidneys and the ablation zone on UE-CT following cryoablation The median attenuation values for normal kidneys and the cryoablation zone were 34.3 HU (IQR = 30.8-35.8) and 47.4 HU (IQR = 43.1-50.0), respectively (P < 0.0001). HU, Hounsfield unit; UE-CT, unenhanced CT

On UE-CT, the median attenuation values for normal kidneys and the cryoablation zone were 34.3 HU (IQR = 30.8-35.8) and 47.4 HU (IQR = 43.1-50), respectively (P < 0.0001; Figure 5).

**Figure 6:**
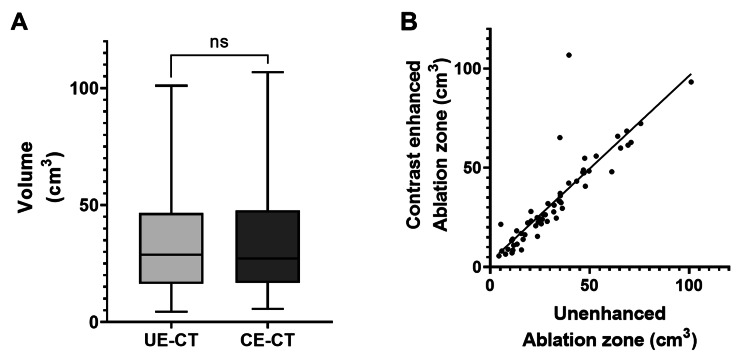
Comparison of the ablation zone as evaluated on UE-CT and CE-CT (A) The median volumes of the cryoablation zone on CE-CT and UE-CT were 27.16 cm³ (IQR = 16.95-47.65, range = 5.6-106.8 cm³) and 28.83 cm³ (IQR = 16.56-45.78, range = 4.4-101.0 cm³), respectively (P = 0.86). (B) These values exhibited a high correlation (r = 0.95; 95% CI = 0.91-0.97). CE-CT, contrast-enhanced CT; UE-CT, unenhanced CT; ns, not significant

The median renal volumes for the unenhanced regions on CE-CT and those exhibiting attenuation changes on UE-CT were 26.52 cm³ (IQR = 17.27-47.65, range = 5.6-106.8 cm³) and 28.83 cm³ (IQR = 16.78-45.78, range = 4.4-101.0 cm³), respectively (P = 0.86, Figure 6A). These values demonstrated a strong correlation (r = 0.95; 95% CI = 0.91-0.97, Figure 6B).

## Discussion

In this study, we successfully estimated the ablation zone using unenhanced UE-CT during the early post-procedure period. Although the contrast with the normal renal parenchyma was lower on UE-CT than on CE-CT, the ablation zone was detectable and highly correlated with CE-CT.

The measurement of the ablation zone showed a high correlation between UE-CT and CE-CT. This finding is particularly important as it suggests that UE-CT could be a viable alternative for volume assessment in patients for whom contrast administration is contraindicated. While CE-CT offered superior boundary delineation, these results underscore the potential of UE-CT as a valuable tool in post-cryoablation assessment. Some literature suggests that unenhanced MRI could be a better alternative [8,9]; however, it is limited by its lower accessibility and spatial resolution, which increases the potential for errors. The ability to accurately assess ablation volumes without contrast provides a safer alternative for at-risk patients, potentially allowing for more frequent and less invasive follow-up protocols. This could lead to improved patient care and earlier detection of treatment failure or recurrence while minimizing the risks associated with repeated contrast exposure.

This study illustrated that the cryoablation zone could be recognized on UE-CT in the early stages after cryoablation. Although the cryoablation zone is reportedly unclear on UE-CT [8,10], the zone exhibited higher attenuation values, and the ablation volume on UE-CT was comparable to that on CE-CT. Prior studies noted the presence of a transitional zone at the ablation margin in early histological examinations of renal freeze ablation in animal experiments. This zone contains both necrotic and viable cells, along with fibrous tubules, glomeruli, hemosiderin deposits, and macrophages [11-14]. In addition, a clinical study reported that relatively high attenuation within the ablation zone can be visualized because of hemorrhage [15]. Conversely, lower attenuation values were observed in the ablation zone on CE-CT, indicating reduced blood flow and tissue necrosis, reflecting the desired outcome of cryoablation [16]. The large median difference in attenuation values suggests that CE-CT is a reliable method for distinguishing ablation zones.

The change in attenuation values in normal kidney tissue before and after cryoablation highlights the impact of this treatment on surrounding healthy tissue. A subtle increase in attenuation values may indicate minor changes in tissue composition or structure following treatment, possibly due to mild edema or inflammation. The study findings suggest that cryoablation effectively targets the tumor while largely sparing surrounding healthy tissue. The minimal impact on surrounding tissue supports the safety of cryoablation as a treatment option for renal tumors.

Limitations

This study had several limitations. First, it was a retrospective study conducted at a single institution with a small sample size. Second, the contrast between the tumor and the surrounding tissue was minimal or absent, making margin evaluation beyond the scope of this research. Third, we excluded patients who underwent preoperative lipiodol marking or postoperative TAE for hemorrhage. Fourth, this study focused on the early period of cryoablation, making it uncertain whether the results would differ during long-term follow-up after cryoablation.

## Conclusions

UE-CT can reasonably estimate the ablation zone in patients with RCC, particularly for those unable to undergo CE-CT. Further research with larger sample sizes is necessary to validate the clinical utility of UE-CT and to assess the reproducibility of the results across diverse cases.
